# Production of Biocomposites Based on Biodegradable Resin, Cellulose, and Lignin Using a Statistical Approach

**DOI:** 10.1021/acsomega.6c02743

**Published:** 2026-07-02

**Authors:** Walber A. Freitas, Rodrigo A. Moreira, João P. F. Amaral, Rodrigo C. B. Batista, Werner Timans, Rudy Folkersma, Katja Loos, José M. E. de Matos

**Affiliations:** † Interdisciplinary Laboratory of Advanced Materials (LIMAV), 67823 Universidade Federal do Piaui, Ministro Petrônio Portella University Campus, Av. Universitária, S/N—Ininga, Teresina, Piauí 64049-550, Brazil; ‡ Circular Plastics, Academy Tech & Design, 84811 NHL Stenden Hogeschool—Locatie Emmen, Van Schaikweg 94, Emmen, Drenthe 7811 KL, Netherlands; § Macromolecular Chemistry and New Polymeric Materials, Zernike Institute for Advanced Materials, 3647 University of Groningen, Groningen, Groningen 9747 AG, Netherlands

## Abstract

This work proposes the use of lignin and cellulose as fillers in the production of composites based on the resin Plantics-GX, a biopolymer commercialized by Plantics B.V., which serves as a sustainable alternative to conventional plastics as it is derived from renewable sources. In the context of the growing demand for environmentally friendly materials, the development of biobased composites represents a key step toward sustainable engineering solutions. A statistical mixture design of experiments was applied to ensure reliable modeling of the formulation and to optimize the component proportions to maximize the composite’s mechanical performance. The tests reported in this paper showed that adding fillers to the Plantics resin increases the mechanical properties of the composite, making it mechanically stronger and more thermally stable, allowing it to be used more securely across a broader range of applications.

## Graphical abstract

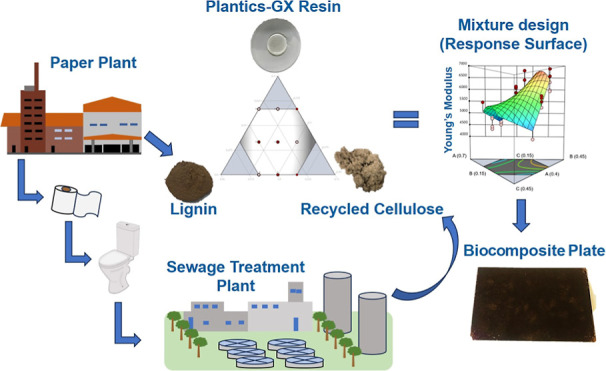

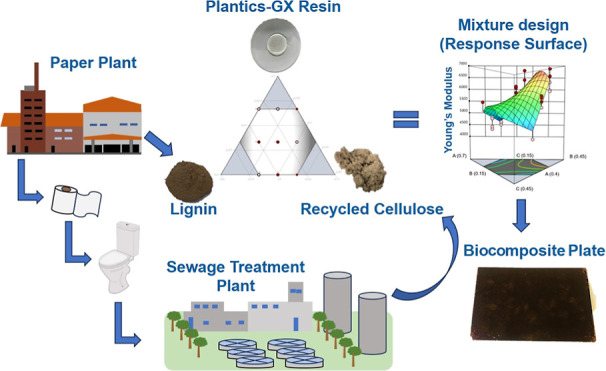

## 1 Introduction

Contemporary global challenges, such as the rapid depletion of fossil fuel reserves and severe environmental pollution caused by the accumulation of petroleum-based plastics, have strongly driven the search for sustainable alternatives.^1–4^ Consequently, there has been a significant increase in research focused on biodegradable plastics (bioplastics), which are derived from renewable sources and capable of mitigating the carbon footprint and ecotoxicological impacts associated with conventional synthetic plastics.^3,4^ The development of these biobased materials is perfectly aligned with the principles of the circular economy, promoting resource efficiency and minimizing waste generation at the end of their useful life.^1,3,5^

In this scenario of transition to cleaner materials, Plantics-GX resin emerges as an ecological and promising alternative in the field of biodegradable polymers.^6^ As an innovative, renewable material, Plantics-GX has considerable potential to replace conventional plastics in a variety of industrial applications.^6^ However, because it is a new biodegradable polymeric matrix, Plantics-GX is still a material little explored in the scientific literature. Currently, there is a lack of research investigating in depth its fundamental properties, processing capacities and true technological, structural and commercial potential.

To circumvent any limitations of biodegradable resins and, at the same time, reduce production costs, the incorporation of natural fillers has been widely adopted and recommended.^7,8^ Cellulose and lignin stand out as ideal filler materials to reinforce this new polymeric matrix of Plantics-GX. Cellulose is the most abundant natural polymer in the world, known for its high crystallinity and excellent mechanical properties, being able to significantly improve the stiffness and tensile strength of polymeric blends.^9–11^ Lignin, in turn, is the second most abundant natural biopolymer.^3,7,8^ It is a three-dimensional aromatic macromolecule that acts as a structural support, providing rigidity and thermal stability, in addition to having great chemical reactivity and acting as a natural antioxidant agent.^12–16^ The joint use of these lignocellulosic biomasses acts synergistically, not only reducing the final cost of the composite, but also accelerating and modulating its biodegradation process and its thermomechanical properties.^17–19^

Mixture design of experiments is a highly efficient statistical approach to understand, predict and optimize complex polymeric matrices.^20^ This method allows for the systematic investigation of cause and effect relationships, evaluating not only the individual influences of each component, but also the synergistic effects between the resin and the filler materials on the final properties of the composite.^21,22^ The application of response surface methodologies allows for a drastic reduction in the number of laboratory experiments, optimizing the processing parameters and identifying the optimal proportions of each constituent to achieve maximum mechanical and thermal performance.^22^

In this sense, the objective of the present study focuses on the application of the response surface methodology and statistical planning of mixtures to produce, model and optimize composites based on the lignin and cellulose-reinforced Plantics-GX resin. The main effort lies in the use of rigorous statistical modeling to generate a reliable predictive model that correlates the composition of the mixture with the thermal, morphological and mechanical characterizations obtained. It is hoped that this optimized model will serve as a solid foundation and guideline for future research involving this new resin, expanding the frontiers of its application and enabling the design of new sustainable and high-performance green plastics.

## 2 Materials and Methods

### 2.1 Materials

Plantics-GX was obtained from Plantics B.V., lignin (*Miscanthus* soda lignin) from WEPA Nederland B.V., and recycled cellulose from ArboCel, and deionized water. The recycled cellulose used in this work was recovered from municipal wastewater treatment plants, originating predominantly from toilet paper residues in the sanitation system, followed by a rigorous sanitization process. All materials and chemicals were used as received without further purification. Silver crest stand mixer, VWR venti-line drying oven, aluminum mold (designed at University of Groningen), Teflon sheets, silicone spray and trays, LabEcon 600 Fontijne Press.

### 2.2 Methods

#### 2.2.1 Elaboration of the Samples

Design of experiments is a statistical technique for analyzing the effects of independent variables (factors) on dependent variables (responses). Unlike other experimental design techniques, mixture design requires the sum of the proportions to equal one, and since it involves a mixture, the factors are no longer independent variables.^23^ A mixture of *q*-components is shown in eq 1.

1 $$0\leq x_{i}\leq 1,i=1,2,...,q,\sum_{i=1}^{q}x_{i}=1=100\%$$

where *x*_*i*_ represents the ratio of the *i*-th component in the mixture. The *q*-components form a regular (*q* – 1)-dimensional simplex. Mixture experiments usually involve additional complications, such as limits on constituent concentrations that must be considered when elaborating a model. A bounded subregion of planning interest is constructed by imposing constraints on the proportions of the components of the mixture. The range of *x*_*i*_ is defined as follows

$$0\leq L_{i}\leq x_{i}\leq L_{s}\leq 1$$

where *L*_*i*_ is the minimum proportion of component *i* in the mixture and *L*_*s*_ is the maximum proportion of component *s* in the mixture.^24,25^

#### 2.2.2 Preparation of the Mixture Design

To investigate the properties of the Plantics-GX resin mixed with different proportions of lignin and recycled cellulose, a series of proportions were prepared in relation to the percentage of mass of Plantics-GX/lignin/recycled cellulose, considering the limits: 0.4 ≤ A: Plantics-GX ≤ 0.6; 0.15 ≤ B: lignin ≤ 0.35; 0.15 ≤ C: cellulose ≤ 0.35 (Figure 1). The restrictions were based on previous laboratory tests, taking into account the resin’s processing. The compositions of the samples are listed in Table 1.

**Figure 1:**
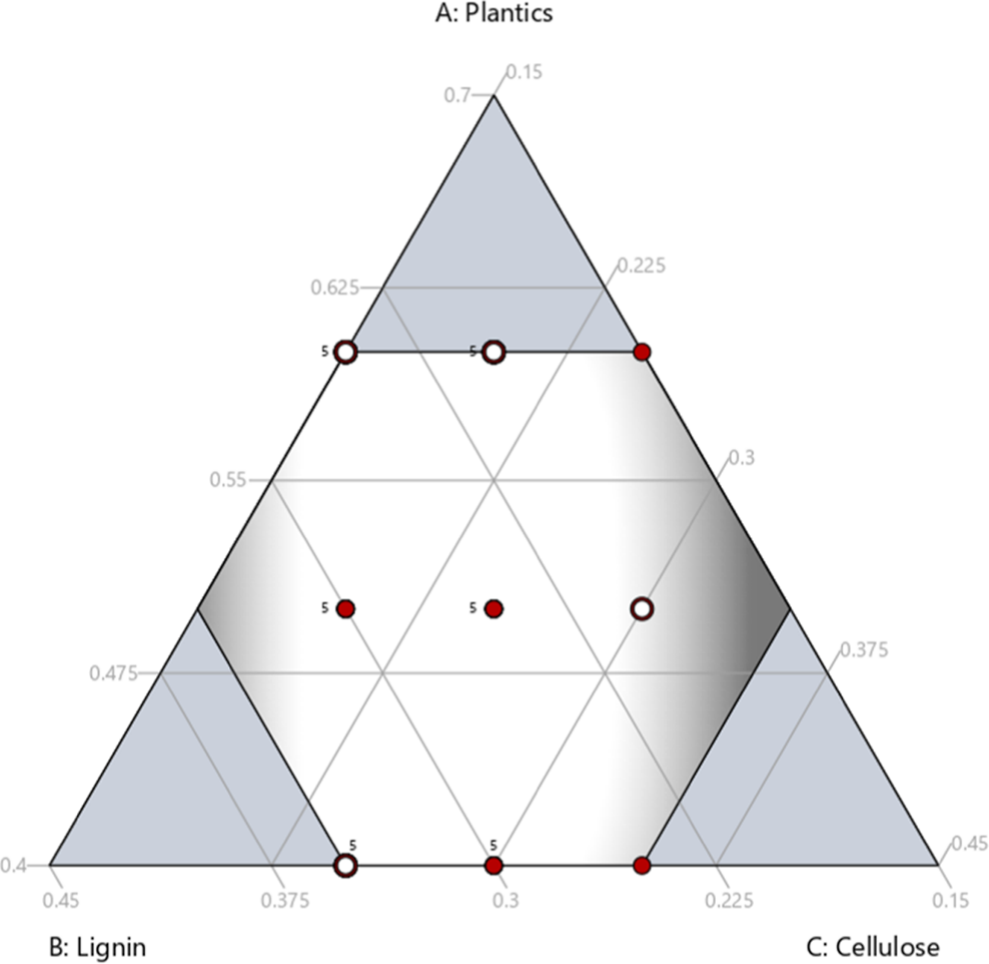
Schematic representation of the pseudomixture with its defined boundaries.

**Table 1:** Mixture Proportions of Plantics-GX, Lignin, and Cellulose

ID	A: Plantics-GX	B: lignin	C: recycled cellulose
NL301	0.6	0.2	0.2
NL302	0.6	0.25	0.15
NL303	0.6	0.15	0.25
NL304	0.5	0.25	0.25
NL305	0.5	0.3	0.2
NL306	0.5	0.2	0.3
NL307	0.4	0.3	0.3
NL308	0.4	0.35	0.25
NL309	0.4	0.25	0.35

Stat-Ease 360 software was used to process data for mixture design, empirical model development, analysis of variance, diagnosis, and optimization. The chosen answer was Young’s modulus, as it is a mechanical property related to a material’s stiffness, defined as the ratio of stress to strain, which reduces the number of answers to analyze and provides a general understanding of the material’s linear elasticity. Six proportions were prepared with five replicates, and three additional proportions (NL303, NL306, and NL309) were prepared to calculate the lack of fit, resulting in 33 runs. Considering the established constraints, the type of design chosen was I-optimal, and the reduced quadratic model (eq 2) was selected

2 $$ŷ=b_{1}^{*}A+b_{2}^{*}B+b_{3}^{*}C+b_{13}^{*}AC$$

where *ŷ* is the value of the response of interest, *b*_1_, *b*_2_ and *b*_3_ are the coefficients of individual factors, *b*_13_ represent the coefficient of two interacting factors, and A (Plantics-GX), B (lignin) and C (cellulose) are the components.^26^

The weight of all the samples amounts to 200 g. After weighing all the components inside a bowl, they were mixed for 1 min using a kitchen machine at maximum speed to ensure high homogeneity and optimal dispersion of the biobased reinforcements within the matrix. After mixing, the samples were stirred with a silicone spatula to avoid clumping at the bottom of the bowl and to ensure that the resin was well mixed with the biobased reinforcements (lignin and cellulose). After that, the sample is mixed for another 2 min at the same speed. Up to 50 mL of deionized water were added to the sample to facilitate the mixing.

#### 2.2.3 Pre-curing

To initiate the precuring stage, the mixture was evenly distributed in a silicone tray and heated in a ventilation oven at 160 °C. The drying process was interrupted after 30 min to manually stir the material, breaking down formed agglomerates, before returning it to the oven for another 30 min. After the 60 min thermal treatment, the precured material was homogenized in a high-speed blender for 2 min at maximum power to ensure a refined powder. The resulting mixture was then transferred to the mold for compression.

#### 2.2.4 Hot-Pressing

The pressing mold consisted of two moving parts: the lid and screwable, removable sides. The inner part of the mold measured 30 by 25 cm and was made entirely of aluminum. Before adding the sample, the mold was sprayed with silicone, and a Teflon sheet was placed on the bottom and sides of the mold. The same was done to the lid before closing, after the samples were evenly placed in the mold.

The mold was then placed in a LabEcon 600 Fontijne Press, with the temperature set to 160 °C. For 10 min, 145 kN of force was applied until the system cooled and the mold was released from the press as shown in Figure 2.

**Figure 2:**
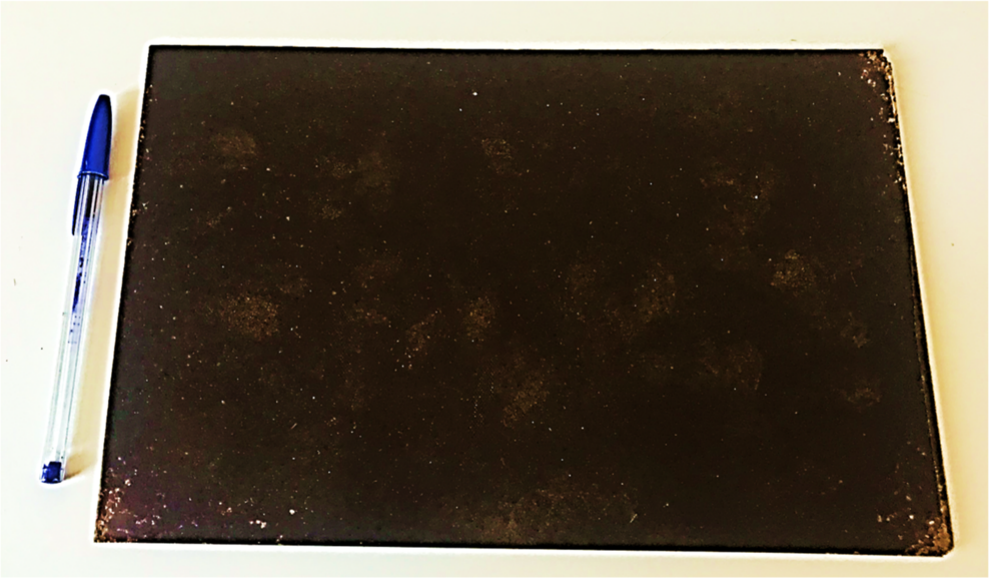
Composite plate after hot-pressing.

#### 2.2.5 Post-curing

Following the compression molding, the solid and uniform plaque was carefully demolded. A subsequent postcuring stage was then conducted by maintaining the sample in a forced-air oven at 100 °C for 24 h. This final thermal treatment was critical for eliminating the residual surface tackiness observed in the demolded plaques, thereby driving the resin cross-linking reaction to completion. Consequently, this step not only provided a defect-free surface finish but also ensured the optimal dimensional stability and structural integrity of the biocomposites prior to characterization.

#### 2.2.6 Cutting

The molded plates were cut into rectangular type 2 specimens measuring 25 × 250 × 2 mm using a saw, according to the ISO 527-4 standard.^27^ The specimens were placed in labeled zip-lock bags and conditioned at 23 °C for an additional 24 h prior to mechanical testing.

#### 2.2.7 Mechanical Tests

Tensile properties were evaluated using a Zwick UPM 14740 ZMART.PRO universal testing machine equipped with a Zwick BZ1-EXZW003 extensometer, according to the ISO 527-4 standard.^27^ The specimens were tested at a crosshead speed of 2 mm/min under a preload of 0.1 MPa and a gauge length of 50 mm. At least five specimens of each formulation were tested. Young’s modulus was calculated from the slope of the initial linear elastic region of the stress–strain curves using the machine’s native automated linear regression function.

#### 2.2.8 X-ray Diffraction (XRD)

The composites’ X-ray diffraction analysis was conducted on a Bruker D8 Advance powder diffractometer to investigate their crystalline structure and phase composition. The samples were scanned over a 2θ range of 5° to 50°.

#### 2.2.9 Thermal-Gravimetric Analysis (TGA)

The thermogravimetric analysis (TGA) was conducted using a TGA5500 under a nitrogen (N_2_) atmosphere at a heating rate of 10 °C/min from 10 to 700 °C, with an isothermal step of 2 min at the end. Analysis of the degradation temperature (*T*_d_) provided information on the samples’ thermal stability and susceptibility to decomposition at different temperatures. The same procedure was also used on a pure Plantics sample to identify the reinforcing agents’ effects on their thermal stability.

#### 2.2.10 Fourier Transform Infrared Spectroscopy (FTIR)

To evaluate the samples, FTIR analysis was performed on a Nicolet Summit Pro spectrometer (Thermo Fisher). Spectra were acquired spanning from 4000 to 400 cm^–1^, utilizing 16 scans per sample and a 0.45 cm^–1^ resolution in attenuated total reflection (ATR) mode.

## 3 Results and Discussion

### 3.1 Analysis of the Experimental Model

The ANOVA (analysis of variance) based on Young’s modulus results (Table 2) was analyzed using the Stat-Ease 360 program to verify the statistical significance of the mixing model. Based on the responses, outliers such as runs 20 and 33 were removed to improve model fit. Table 3 compiles these results, showing not only the significance of the model but also those of the components A, B and C, which individually and together influence (interaction) the response variables.

**Table 2:** Runs of Mixture Design

		mixture variables			response
ID	run	A: Plantics-GX	B: lignin	C: cellulose	Young’s modulus (MPa)
NL302	1	0.6	0.25	0.15	4640
NL305	2	0.5	0.3	0.2	5650
NL308	3	0.4	0.35	0.25	6100
NL308	4	0.4	0.35	0.25	6460
NL303	5	0.6	0.15	0.25	5650
NL301	6	0.6	0.2	0.2	4540
NL304	7	0.5	0.25	0.25	6230
NL308	8	0.4	0.35	0.25	6990
NL304	9	0.5	0.25	0.25	6040
NL304	10	0.5	0.25	0.25	5460
NL304	11	0.5	0.25	0.25	6290
NL307	12	0.4	0.3	0.3	5770
NL302	13	0.6	0.25	0.15	5100
NL307	14	0.4	0.3	0.3	5910
NL304	15	0.5	0.25	0.25	6230
NL302	16	0.6	0.25	0.15	4600
NL308	17	0.4	0.35	0.25	6140
NL305	18	0.5	0.3	0.2	6860
NL305	19	0.5	0.3	0.2	5660
NL305	20	0.5	0.3	0.2	7950
NL301	21	0.6	0.2	0.2	4880
NL305	22	0.5	0.3	0.2	6480
NL301	23	0.6	0.2	0.2	4440
NL307	24	0.4	0.3	0.3	6000
NL307	25	0.4	0.3	0.3	5540
NL301	26	0.6	0.2	0.2	4930
NL301	27	0.6	0.2	0.2	4880
NL302	28	0.6	0.25	0.15	4910
NL302	29	0.6	0.25	0.15	4510
NL309	30	0.4	0.25	0.35	4410
NL306	31	0.5	0.2	0.3	5436
NL308	32	0.4	0.35	0.25	6700
NL307	33	0.4	0.3	0.3	3560

**Table 3:** Analysis of Variance (ANOVA) for the Responses

source	sum of square	df^a^	mean square	*F*-value	*p*-value	
**model**	1.427 × 10^7^	3	4.756 × 10^6^	36.36	<0.0001	significant
linear mixture	8.456 × 10^6^	2	4.228 × 10^6^	32.33	<0.0001	
AC	5.811 × 10^6^	1	5.811 × 10^6^	44.43	<0.0001	
**residual**	3.531 × 10^6^	27	1.308 × 10^5^			
lack of fit	8.234 × 10^5^	5	1.647 × 10^5^	1.34	0.2853	not significant
pure error	2.708 × 10^6^	22	1.231 × 10^5^			
**Cor total**	1.780 × 10^7^	30				
***R*** ^**2**^						0.8016
**adjusted *R*** ^**2**^						0.7796
**predicted *R*** ^**2**^						0.7355
**adequate precision**						14.8216

a df = degree of freedom.

Table 3 shows the significance of the model, as well as the effects of a linear combination of individual parameters and the AC interaction effect. Interactions with nonsignificant effects were removed from the model in order to improve its prediction and reduce the VIFs (variance inflation factor). An *F*-value higher than the tabulated values indicates that the variation described by the model is significantly greater than the variation inherent to the process. Thus, the *p*-value is given as the probability of reaching the *F*-value. A value <0.05 indicates a statistically significant difference between the means. Thus, the model presented can be considered satisfactory.^25^ The lack-of-fit criterion in a model indicates whether the experimental data are well fit. A high *p*-value (*p* > 0.05) suggests that the residual variation is not significantly greater than the experimental error. Therefore, the square mean of the lack of fit and the square mean of the pure error should reflect only the random errors inherent to the system under study. In other words, there is no need for adjustment, and the model has adequate complexity to describe the process.^28^

The value of *R* ^2^ = 0.8016 (coefficient of determination) represents the fraction of the variation explained by the regression or by the model coefficients, i.e., although this coefficient measures how well the model fits the experimental data, it is not suitable for assessing lack of fit, as it does not consider the number of degrees of freedom used to determine the model, therefore this value alone does not guarantee that the model is correct; analysis of other variables and parameters is necessary to validate the model.^23,28^ The predicted *R* ^2^ of 0.7355 agrees with the adjusted *R* ^2^ of 0.7796, considering that the difference between them is less than 0.2. Adequate Precision measures the signal-to-noise ratio. A ratio higher than 4 is desirable. Its ratio of 14.8216 indicates a suitable signal; thus, model can be used to navigate the space of interest.^25^ The coefficient of variation (CV %) was used to validate the accuracy of the treatment, helping to identify the level of dispersion of the data in relation to the mean. CV % values equal to or less than 10% indicate greater experimental reliability, and for this model, the CV % value was 6.46%.^23^

Figure 3a shows the residuals obtained in the graph for Young’s modulus. It is noted that the internally studentized residues tend to follow the normal distribution. By definition, these studentized residuals indicate the difference between the actual and predicted values, expressed in standard deviations (estimated from the residuals themselves).^23^ The straight line in this graph, without clusters in the data, suggests that the residuals were randomly distributed and had constant variance.^25,29^ The model’s diagnosis is complemented by the analysis of Figure 3b–d. Figure 3b shows the residuals as a function of execution order revealing no trend or grouping that suggests dependence on the experimental sequence. To evaluate the influence of individual observations, the Cook’s distance (Figure 3c) was plotted, a parameter that estimates the change in the model’s parameters when excluding a given run, helping to identify outliers. Figure 3c shows uniform data and no outliers, considering that no data exceeded Cook’s distance value (0.860614).^23,25^ Figure 3d illustrates the relationship between the actual responses and the responses predicted by the model.

**Figure 3:**
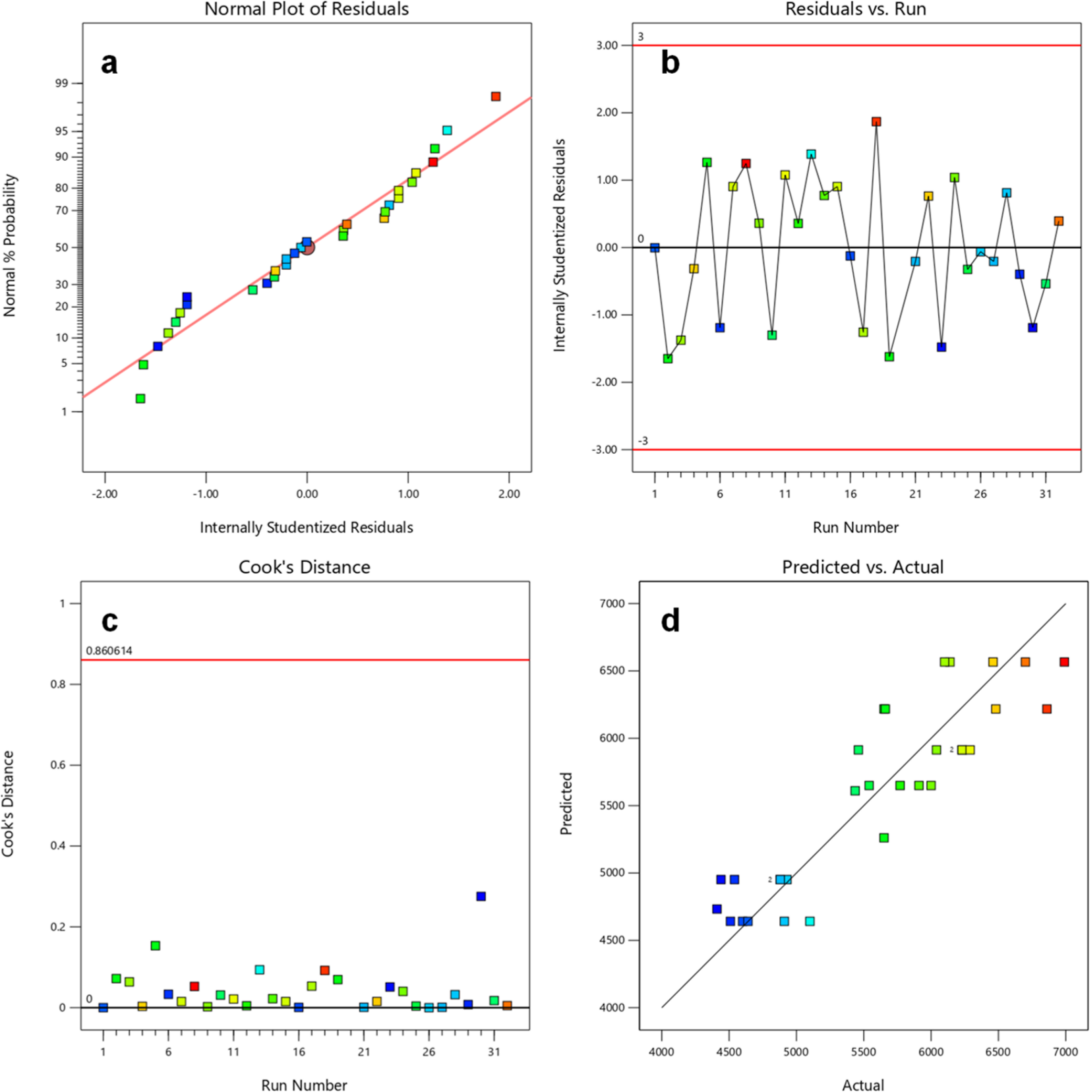
Normal probability graph of the residuals (a); relationship between the residues and the order of execution (b); Cook’s distance (c); relationship between the predicted data and the actual data (d).

### 3.2 Optimization

After applying the ANOVA test and analyzing the diagnostic graphs, the model demonstrated representativeness and strong predictive capacity. The final model, including the numerical values of the respective coefficients, is presented in Table 4 and eq 3.

**Table 4:** Coefficients in Terms of Coded Factors

component	coefficient estimate	df^a^	standard error	95% CI^b^ low	95% CI^b^ high	VIF
A-Plantics	2761.32	1	291.33	2163.57	3359.07	3.89
B-lignin	8400.73	1	359.03	7664.06	9137.41	5.53
C-cellulose	2898.21	1	504.01	1864.06	3932.35	6.15
AC	11,044.69	1	1656.96	7644.89	14,444.48	4.47

a df = degree of freedom.

b CI = confidence intervals.

The coefficient estimate expresses the expected variation in response for each unit change in a factor, provided the others remain constant. In orthogonal designs, the intercept acts as the overall mean of all trials, while the coefficients serve as adjustments to this mean based on the factor settings. In perfectly orthogonal systems, the VIF equals 1. Higher values indicate the presence of multicollinearity, with indices below 10 being commonly accepted in practice. It should be noted that, in mixture designs, there is no orthogonality, since the variables are intrinsically dependent, some degree of multicollinearity is expected. Despite this, the correlation between the predictor variables does not, in general terms, prevent obtaining a good statistical fit. Multicollinearity does not usually compromise inferences about response means or the accuracy of predictions for new data; its main impact is the difficulty of mathematically isolating the individual contribution of each correlated factor.^30,31^

3 Young’smodulus=2761.32×A+8400.73×B+2898.21+11,044.69×AC

The equation in terms of coded factors can be used to predict responses to specific levels of each factor. The coded equation is useful for identifying the relative impact of the factors by comparing the factor coefficients.^32^ This equation reflects the dependence of the mixture’s Young’s modulus on its composition, subject to the specific processing conditions used in this study.

From the response surface and contour plots (Figure 4), it can be observed that the formulations with higher lignin content showed a higher Young’s modulus; Since cellulose also generates a slight positive response in the material’s stiffness, evidenced by the saddle-shaped response surface. Therefore, for the calculation of the prediction point and validation of the model, the following criteria were defined in the software used: maximize the amount of Plantics-GX, lignin, and cellulose; set the importance level of each component to 4. According to the model elaborated, the design expert program suggested as the optimal point the proportion of 50% Plantics-GX (A), 25% lignin (B) and 25% cellulose (C) the same of NL304 sample.

**Figure 4:**
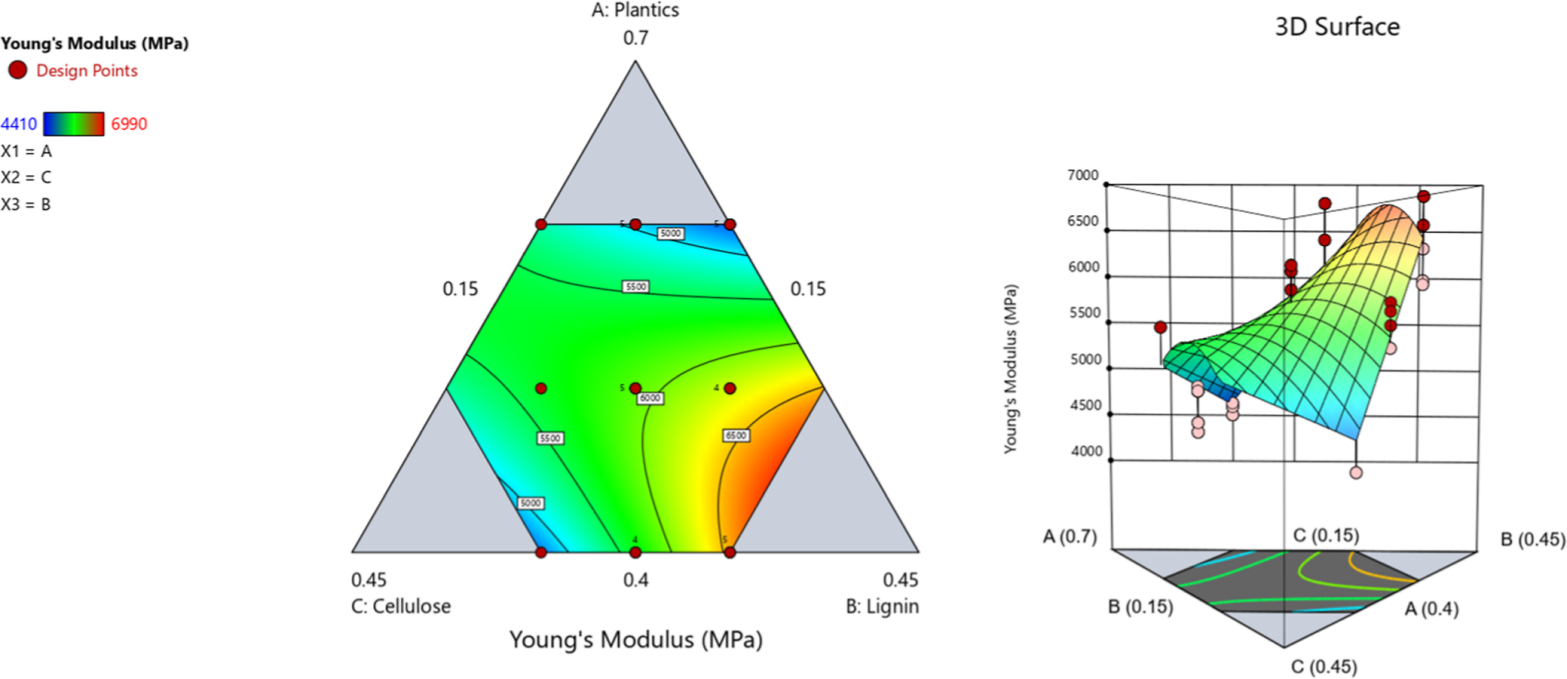
Graphics of contour area and response surface.

To verify the ideal proportion generated by the software, the results were verified by the relative standard error (RSE) (eq 4), where ten runs were performed to verify the model (Table 5).

4 $$RSE,\%=\frac{\left(actual\;value-predicted\;value\right)}{predicted\;value}\times 100$$

**Table 5:** Races to Model Validation

		observed		
response	predicted value (MPa)	run	response (MPa)	mean (MPa)
Young’s modulus (MPa)	5962.87	1	6220	5993.4 ± 435
		2	5210	
		3	5480	
		4	6140	
		5	6710	
		6	5810	
		7	5970	
		8	6030	
		9	6434	
		10	5930	

The predicted and experimental Young’s modulus values were 5963 and 5993 MPa, respectively, under optimal conditions. The comparison between the experimental and predicted results indicated an error of 0.51%. Demonstrating that, for the conditions and formulations adopted in this study, it resulted in a satisfactory model with good predictive capacity.

### 3.3 Mechanical Properties

The implementation of specific thermal treatments, both before and after compression molding, was crucial for optimizing the final quality of the biocomposites. The incorporation of a precuring step at 160 °C prior to compression was essential, as preliminary trials without this thermal treatment resulted in excessive formation of voids and bubbles during hot-pressing. This precuring stage facilitated the removal of entrapped air and volatiles, stabilizing the resin and leading to a more homogeneous matrix. Furthermore, a postcuring stage at 100 °C for 24 h proved critical after demolding. Initial observations of the demolded plaques indicated a residual surface tackiness. This subsequent thermal treatment drove the cross-linking reaction of the resin to completion, effectively eliminating the tackiness. Consequently, the postcuring step not only provided a defect-free surface finish but also ensured the optimal dimensional stability and structural integrity of the samples for subsequent characterizations.

The mechanical performance of the Plantics-GX/lignin/cellulose biocomposites was evaluated to determine how the formulation ratios influence the material’s stiffness and strength. By applying the statistical mixture design, it was possible to assess the complex synergistic and antagonistic interactions between the biobased reinforcements and the resin matrix. The average results for Young’s modulus (*E*_T_), tensile stress (σ_M_1__), and strain (ε_M_1__) for each experimental ratio are summarized in Table 6.

**Table 6:** Young’s Modulus (*E*_T_), Stress (σ_M_1__) and Strain (ε_M_1__) Values for Each Strip

	*E*_T_ (MPa)	σ_M_1__ (MPa)	ε_M_1__ (%)
NL301	4750 ± 464	6.90 ± 1.67	0.153 ± 0.04
NL302	4590 ± 460	7.41 ± 1.48	0.175 ± 0.02
NL303	5650 ± 518	9.86 ± 1.82	0.156 ± 0.08
NL304	5860 ± 558	8.59 ± 1.89	0.130 ± 0.02
NL305	6230 ± 1110	10.04 ± 3.26	0.160 ± 0.04
NL306	5430 ± 844	7.3 ± 1.41	0.130 ± 0.03
NL307	5360 ± 1020	7.69 ± 3.14	0.140 ± 0.05
NL308	6560 ± 774	6.48 ± 1.57	0.10 ± 0.03
NL309	4410 ± 567	1.89 ± 1.65	0.03 ± 0.03

Although samples NL308 and NL305 exhibited the highest absolute Young’s modulus values, the statistical optimization identified sample NL304 as the optimal formulation. While lignin acts as a reinforcing agent due to its rigid aromatic macromolecules, high concentrations can lead to increased brittleness.^33^ This effect was observed in sample NL308, which displayed poor elongation at break (0.10%). In contrast, sample NL304 offered a superior balance, maintaining high stiffness (5860 MPa) with better elongation (0.13%) than the high-lignin samples, validating it as the most robust composition for practical application. It was noted that samples containing 30% (wt) of cellulose and up had the worst mechanical properties, including elongation.

The table also shows that samples with a higher content of Plantics-GX had a bigger elongation at break than the ones that had more biobased reinforcements added to them, presumably because at a higher content, the reinforcing agents are more difficult to disperse uniformly,^34^ forming agglomerates and, consequently, preferential paths that increase the material’s ductility. Also, as Plantics is hydrophilic, it is expected that hydrophobic components such as lignin could be more difficult to disperse in its matrix. Therefore, both the morphology of the biobased reinforcements and their dispersion state in the matrix played an important role in determining the overall mechanical properties of the composites.

Considering the mean and standard deviation values of Young’s modulus of the samples, a descriptive statistical analysis was performed (see Tables S1–S5, Supporting Information) using Jamovi software (version 2.5), which demonstrated that the different formulations evaluated exert a significant effect on the stiffness of the material (Welch’s ANOVA: *F*-value (8, 18.8) = 6.97; *p* < 0.001). The assumptions of normality (Shapiro–Wilk: *W* = 0.979; *p* = 0.416) and homogeneity of variances (Levene: *F*-value (8, 47) = 1.12; *p* = 0.368) were also verified. As the *p*-value is greater than the significance level (0.05) for both, we can assume that the data follow a normal distribution and that the variances between the groups are homogeneous.^35^

The Games–Howell multiple comparisons test (see Table S6, Supporting Information) revealed the formation of two distinct mechanical profiles, isolating the formulations that confer greater stiffness from those that result in less stiff materials. The group with the highest resistance to deformation was led by samples NL308 (mean = 6553) and NL304 (mean = 5860). Sample NL308 showed robust statistical superiority in relation to the less stiff formulations such as NL309 (*p* = 0.003), NL302 (*p* = 0.004) and NL301 (*p* = 0.007). Similarly, sample NL304 was significantly superior to the same samples: NL309 (*p* = 0.021), NL302 (*p* = 0.028) and NL301 (*p* = 0.046). Thus, it can be concluded that samples NL309, NL302, and NL301 established the lower limit of stiffness, registering the lowest average values in the study. The intermediate samples (such as NL305) showed high internal dispersion, not differing statistically from the extremes of the study.

Bonnet Martin et al.^36^ conducted a study on additive manufacturing of thermosetting polymers incorporating at least one biobased constituent, either as a filler or as the thermosetting polymer itself. These fillers are mainly derived from woody biomass, cellulose, and lignin powder. Table 7 lists some biobased thermosetting resins. The Young’s modulus of epoxy resins can range from 0.37 to 3700 MPa,^36^ and given that the biocomposites produced reached an *E*_T_ of 6560 MPa, resins such as Plantics-GX reinforced with biobased materials show great potential for future research.

**Table 7:** Some Bio-Based Thermosetting Polymers^a^

biobased feedstock	primary functional group	layer thickness (μm)	Young’s modulus (MPa)
levoglucosan from cellulosic biomass	thiol and alkene	200	14.49 ± 0.58
commercial bioepoxy	epoxy	1300	1025 ± 130
lignin	acrylate and methacrylate	26–50	480 ± 10
cellulose-derived levoglucosenone	thiol and alkene	50	4.2 ± 0.7
levoglucosan from cellulosic biomass	thiol and alkene	100	12.3 ± 1.0
tartaric acid from grapes	methacrylate	100–500	1244.2

a Source: adapted from Bonnet Martin et al.^36^

## 4 Characterization

### 4.1 XRD

XRD analysis of cellulose and lignin (Figure 5) reveals a significant difference between them. This is due to the highly organized nature of cellulose and the highly disordered nature of lignin. Due to strong hydrogen bonds and van der Waals forces, cellulose exhibits a semicrystalline structure. These intermolecular forces are responsible for organizing the chain, generating specific diffraction patterns in XRD.^37^ In the XRD graph of cellulose, the peaks at 2θ angles of approximately 16.5°, 22.5°, and 35° are associated with specific crystallographic planes, such as (110), (200), and (004), respectively, confirming its monoclinic structure. When compared with reference standards, these peaks indicate the presence of type I cellulose.^38–40^ On the other hand, lignin, being a polymer with a branched three-dimensional structure, does not produce defined diffraction peaks in the XRD spectrum. Pure lignin exhibits broad scattering covering a range of 10° to 30°, with its maximum intensity at 2θ = 21.5°; this zone can be related to amorphous carbon peaks.^41^

**Figure 5:**
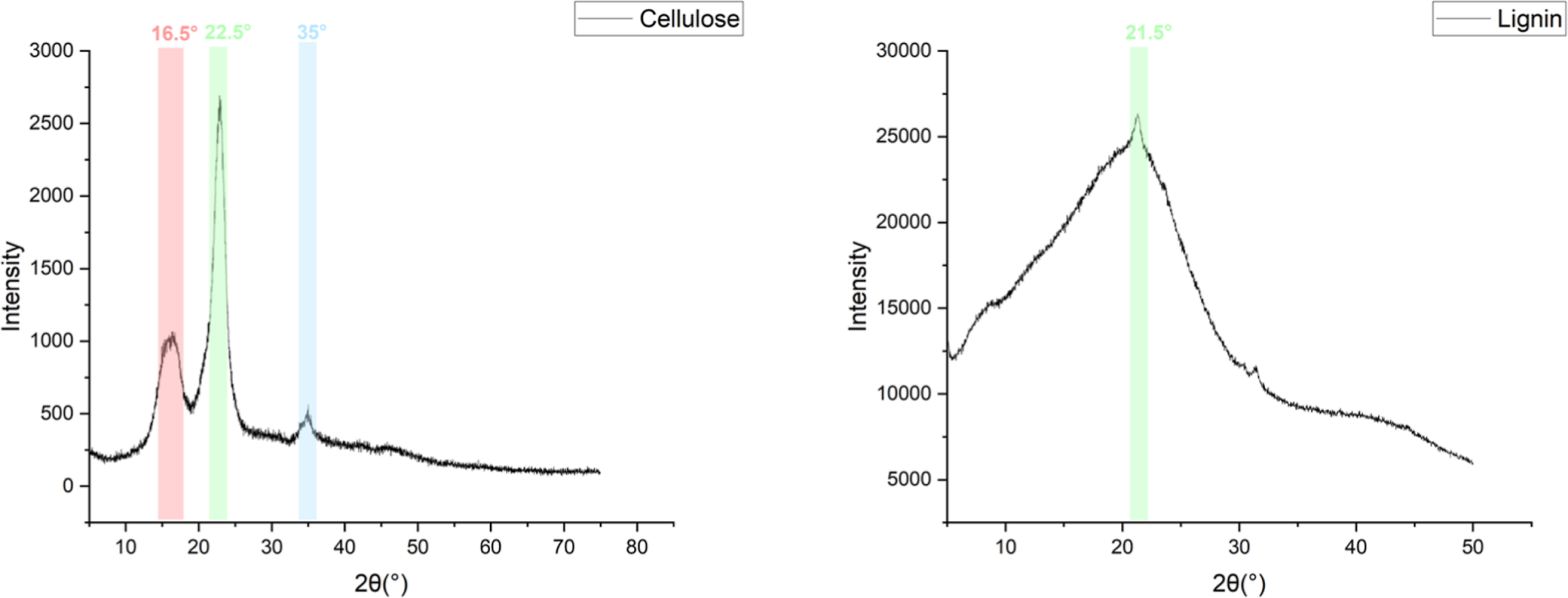
XRD spectra of cellulose and lignin, respectively.

The X-ray diffraction patterns of the samples are shown in Figure 6, revealing distinct diffraction peaks at specific 2θ angles, indicative of the crystallographic planes present in the composite. Although broader peaks were observed, their intensities were not higher around the 2θ angles of 16.5° and 22.5°. The intensities of the diffraction peaks provided information about the preferred orientation and crystallite size of the phases in the samples.^40^ Furthermore, the absence of distinct peaks observed throughout the analysis is attributed to Plantics-GX and lignin, since these two components of the sample do not have the crystalline structure of cellulose and are amorphous, conferring a more amorphous characteristic to the composites. As the biobased reinforcements increase in the composite, there is greater definition of the peaks. The first two samples presented the most distinct profiles due to their higher Plantics-GX content, exhibiting wider peaks that overlap those of cellulose and highlight their amorphous structure due to the three-dimensional network of the resin.^6^

**Figure 6:**
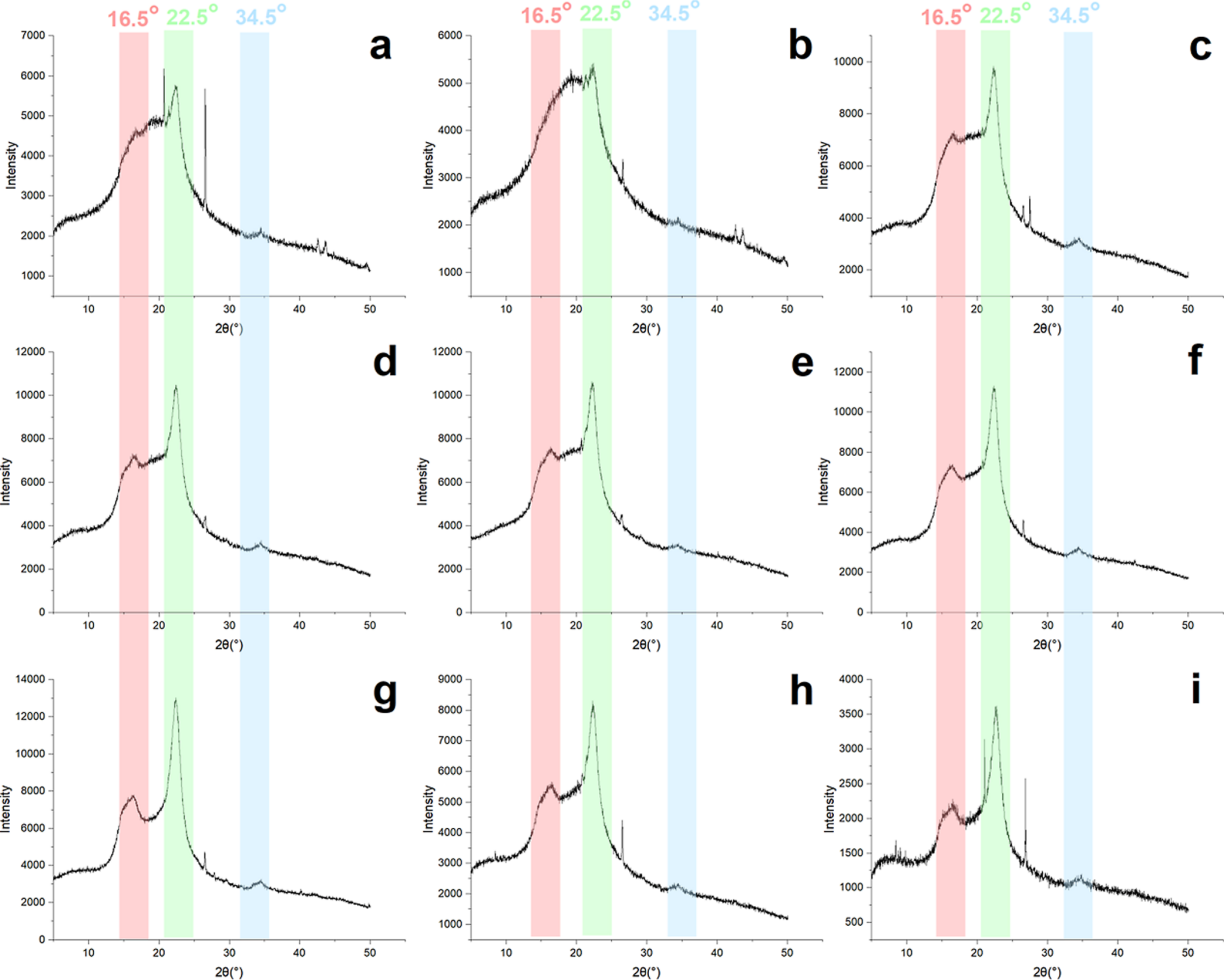
XRD of samples: NL301 (a), NL302 (b), NL303 (c), NL304 (d), NL305 (e), NL306 (f), NL307 (g), NL308 (h) and NL309 (i).

In XRD, it was observed that the crystalline integrity of the cellulose was maintained during the processing of the composite. This was evidenced by the preservation and sharpening of its characteristic peaks with increasing filler proportions, confirming that its original crystalline structure (cellulose I) was not destroyed during interaction with the resin and lignin.^9,42^ It also showed no formation of crystalline structures, inferring that the interaction between the reinforcements and the matrix is of an interfacial physicochemical nature (such as van der Waals forces and hydrogen bonds), and no chemical reaction occurred capable of producing a new compound or new crystallographic planes.

XRD is essential to measure the proportion of crystals present in the sample; therefore, the crystallinity index was calculated using eq 5.

5 $$C_{rl}=\frac{\left(I_{máx}-I_{\min}\right)}{I_{máx}}\times 100$$

The highest and lowest intensities are observed between the two highest peaks (16.5° and 22.5°, respectively). The average crystallinity index was 51%, similar to those reported by Mwaikambo and Ansell^43^ and Jonoobi et al.^44^ when testing cellulose fibers of different sources. Variation in the proportions and crystallinities of cellulose, lignin, and resin is fundamental to achieving a balance between structural strength and flexibility in biocomposites. Since cellulose is highly organized (semicrystalline) and lignin and Plantics-GX are completely disordered (amorphous), the interaction and proportion between them directly and profoundly affect the material’s mechanical performance.

### 4.2 Thermogravimetric Analysis

Figure 7 shows the plots of the percentage weight loss of the samples with the increased temperature.

**Figure 7:**
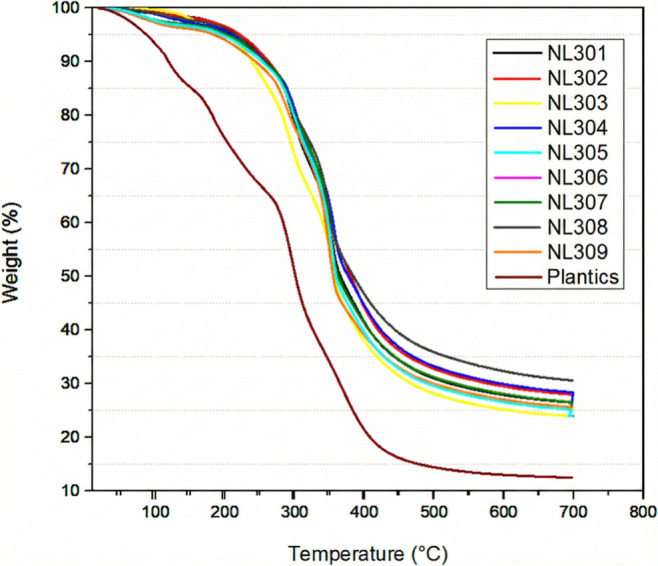
TGA of the all samples and Plantics-GX.

It was noted that Plantics had poor thermal stability, with the lowest *T*_d_ among all the samples tested. The samples with the lowest relative reinforcement content, such as NL301 and NL302, had the highest *T*_d_, indicating that adding these biobased materials improved the resin thermal stability. By adding biobased reinforcements, the behavior changed with the reinforcing agents ratios. Samples that contained more lignin than cellulose had a higher *T*_d_, which can be due to the complex nature of the thermal decomposition of lignin since part of the system’s energy is absorbed to form new bonds within the structure of the macromolecules, and the cleavage of waxes and oligomers.^45^ It is believed that the phenolic groups of lignin can also act as free radical scavenger during TGA, retarding the thermal degradation of the polymer^33^ and making samples with higher lignin content more thermally resistant. The same was not seen in samples containing larger percentages of cellulose.

### 4.3 FTIR Analysis

Figure 8 shows the FTIR analysis of the 9 biocomposites presented in this paper. Notably, all the spectra have the same peaks and, therefore, have the same functional groups. The groups observed in the bio composites’ spectra were related to their components, such as lignin, cellulose, and the Plantics-GX resin made from citric acid and glycerol.^6^

**Figure 8:**
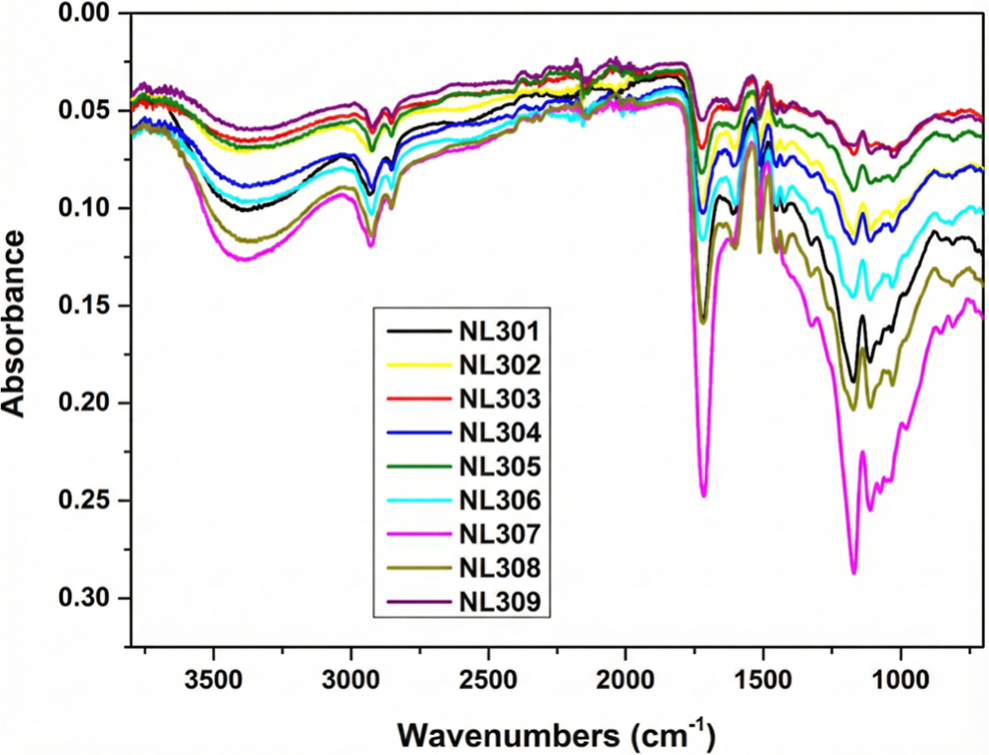
FTIR spectrum of the 9 biocomposites.

The spectral analysis was performed on Figures 8 and 9, and divided into two parts: the section between 3800 and 2000 cm^–1^ and the second part between 2000 and 700 cm^–1^.

**Figure 9:**
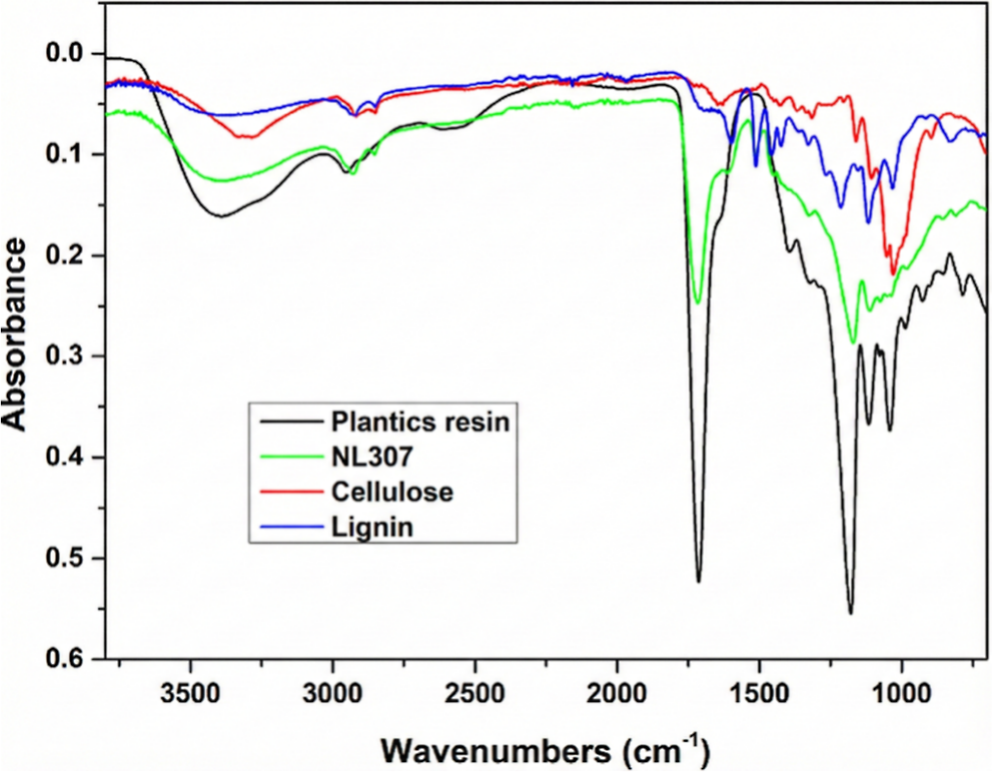
FTIR spectrum of raw materials and sample NL307.

Figure 9 shows that the composite has peaks corresponding to the raw materials individually. In the first part, the peak around 3288 cm^–1^ is related to the O–H stretching, and both peaks around 2930 and 2853 cm^–1^ come from C–H stretching, originating from primary, secondary, and tertiary carbons.^46,47^ These peaks originated from all the biocomposites’ precursors, as they all contain the aforementioned OH and CH groups.^47,48^

In the second part, a peak that is attributed to C=O bonds is present at 1717 cm^–1^, which might come from ester or carboxyl groups present in the resin or the union between the peaks (ester C=O bonds, usually on 1750–1735 cm^–1^ and carboxyl C=O bonds, usually on 1710 cm^–1^).^46,49^ Next to the high peak around 1700 cm^–1^, there is a shoulder, with a weak peak at 1614 cm^–1^, which can be attributed to the C=C aromatic skeleton bonds presented in lignin and the absorption of water by the composite and its components.^48,50^ The next peak, on 1515 cm^–1^ could be associated with the aromatic skeletal vibration of lignin.^47^ Aromatic groups are responsible for giving rigidity to the polymeric matrix, what may justify the increase in Young’s modulus for biocomposites. The peaks from 1300 to 1075 cm^–1^ can all be attributed to C–O bonds, originating from carboxylic acids, esters, ethers, alcohols, and phenols, presented in various components of the biocomposites.^47–51^

Through FTIR analysis of the composites and raw materials, it was observed that no bands disappeared or formed, corroborating the XRD analysis, which showed that no chemical reaction occurred between the constituents of the biocomposite. It is concluded that the interfacial interaction of the components is mainly due to van der Waals forces and hydrogen bonds, evidenced by discrete shifts in the bands corresponding to carbonyl and hydroxyl groups, as well as changes in their intensities.^46–49^

## 5 Conclusions

The proposed statistical model proved to be satisfactory for the conditions adopted in this study for the formulation of a resistant composite. It was possible to investigate the effects of the biobased reinforcements on the resin due to mechanical properties and thermal stability. XRD analysis allowed us to calculate the crystallinity index of the recycled cellulose as 51%. The addition of the reinforcing agents increased the stiffness, as shown in the Young’s modulus of each sample tested. The statistical model validated sample NL304 (50% Plantics-GX, 25% lignin, and 25% cellulose) as the optimal formulation. Although samples with higher lignin content, such as NL308, exhibited slightly higher stiffness, they suffered from reduced elongation at break. The optimized composition (NL304) provides the most effective compromise, maximizing the reinforcing effect of the biobased reinforcements while maintaining sufficient structural integrity and processability suitable for engineering applications. The TGA analysis showed a significant increase in the degradation temperature compared to the pure resin, indicating that using biobased reinforcements is an effective low-cost way to improve the material’s thermal stability.

## Supplementary Material



## Notes

Table S1: data for descriptive statistics analysis. Table S2: one-way ANOVA (Welch’s). Table S3: group descriptives. Table S4: normality test (Shapiro–Wilk). Table S5: homogeneity of variances test (Levene’s). Table S6: Games–Howell post-hoc test—Young’s modulus (PDF)
