# Supplementary material for: Production of Biocomposites Based on Biodegradable Resin, Cellulose, and Lignin Using a Statistical Approach
Source: ACS Omega. 2026 Jul 2;11(28):42263–74. doi: 10.1021/acsomega.6c02743 (PMC13393198; doi:10.1021/acsomega.6c02743)
Supplement: Supplementary file 1 [file ao6c02743_si_001.pdf]

Production of biocomposites based on biodegradable resin, cellulose, and lignin using a statistical approach

Walber A. Freitas,[1,2,3] Rodrigo A. Moreira,[1] João P. F. Amaral,[2] Rodrigo C. B. Batista,[2] Werner Timans,[2] Rudy Folkersma,[2] Katja Loos,[3] and José M. E. Matos[1]*

1. Universidade Federal do Piaui, Interdisciplinary Laboratory of Advanced Materials (LIMAV)

Ministro Petrônio Portella University Campus, Av. Universitária, S/N – Ininga

Teresina, PI, BR 64049-550

2. NHL Stenden Hogeschool - Locatie Emmen, Circular Plastics, Academy Tech & Design

Van Schaikweg 94

Emmen, DR, NL 7811 KL

3. University of Groningen, Macromolecular Chemistry and New Polymeric Materials

Zernike Institute for Advanced Materials

Groningen, GR, NL 9747 AG

*Corresponding author's e-mail: jmematos@ufpi.edu.br

Table S1: Data for descriptive statistics analysis

Samples	Young's Modulus (MPa)	Residuals
NL301	4540	-212.49999999997812
NL301	4880	127.49999999999662
NL301	4440	-312.50000000000415
NL301	5440	687.4999999999976
NL301	4930	177.4999999999982
NL301	3880	-872.5000000000048
NL301	4880	127.49999999999724
NL301	5030	277.49999999999727
NL302	4640	53.33333333333269
NL302	5100	513.3333333333334
NL302	4600	13.333333333333396
NL302	4910	323.33333333333337
NL302	4510	-76.66666666666664
NL302	3760	-826.6666666666667
NL303	5130	-521.6666666666664
NL303	5700	48.33333333333351
NL303	6060	408.33333333333377
NL303	5010	-641.6666666666665
NL303	5660	8.333333333333503
NL303	6350	698.3333333333337
NL304	6230	369.99999999999994
NL304	6040	180.00000000000006
NL304	5460	-400.00000000000006
NL304	4910	-950.0000000000001
NL304	6290	429.99999999999994
NL304	6230	369.99999999999994
NL305	5650	-583.3333333333331
NL305	6860	626.666666666667
NL305	4800	-1433.333333333333
NL305	5660	-573.3333333333331
NL305	7950	1716.666666666667
NL305	6480	246.66666666666703
NL306	6830	1493.3333333333333
NL306	4540	-796.6666666666671
NL306	5400	63.33333333333317
NL306	5220	-116.66666666666686
NL306	5190	-146.66666666666683
NL306	4840	-496.66666666666697
NL307	5770	413.99999999999994
NL307	5910	553.9999999999999
NL307	6000	643.9999999999999
NL307	5540	184
NL307	3560	-1796
NL308	5530	-1022.8571428571431
NL308	7950	1397.142857142857
NL308	6100	-452.85714285714306
NL308	6460	-92.85714285714289
NL308	6990	437.14285714285705
NL308	6140	-412.85714285714306
NL308	6700	147.1428571428571
NL309	3652	-757.5000000000001
NL309	3955	-454.50000000000034
NL309	4258	-151.50000000000009
NL309	4561	151.49999999999997
NL309	4864	454.5000000000001
NL309	5167	757.5000000000001

Table S2: One-Way ANOVA (Welch's)

	F	df1	df2	p
Young's Modulus	6.97	8	18.8	< .001

df=degree of fredom

Table S3: Group Descriptives

	Samples	N	Mean	SD	SE
Young's Modulus	NL301	8	4753	466	165
	NL302	6	4587	461	188
	NL303	6	5652	518	211
	NL304	6	5860	557	228
	NL305	6	6233	1107	452
	NL306	6	5337	794	324
	NL307	5	5356	1019	456
	NL308	7	6553	774	293
	NL309	6	4410	567	231

SD= Standard deviation; SE= Standard error

Table S4: Normality Test (Shapiro-Wilk)

	W	p
Young's Modulus	0.979	0.416

Note. A low p-value suggests a violation of the assumption of normality

Table S5: Homogeneity of Variances Test (Levene's)

	F	df1	df2	p
Young's Modulus	1.12	8	47	0.368

Table S6: Games-Howell Post-Hoc Test – Young's Modulus

		NL301	NL302	NL303	NL304	NL305	NL306	NL307	NL308	NL309
**NL301**	Mean difference	—	166	-899	-1108 *	-1481	-584	-603.5	-180 **	343

Table S6: Games-Howell Post-Hoc Test – Young's Modulus

		NL301	NL302	NL303	NL304	NL305	NL306	NL307	NL308	NL309
	t-value	—	0.664	-3.36	-3.94	-3.08	-1.607	-1.245	-5.36	1.208
	df	—	11.0	10.22	9.70	6.34	7.55	5.06	9.58	9.59
	p-value	—	0.998	0.102	0.046	0.201	0.780	0.913	0.007	0.936
**NL302**	Mean difference		—	-1065	-1273 *	-1647	-750	-769.3	-196 **	177
	t-value		—	-3.76	-4.31	-3.365	-2.00	-1.560	-5.65	0.594
	df		—	9.87	9.66	6.68	8.02	5.35	9.94	9.60
	p-value		—	0.058	0.028	0.141	0.578	0.796	0.004	0.999
**NL303**	Mean difference			—	-208	-582	315	295.7	-901	1242 *
	t-value			—	-0.67	-1.16	0.814	0.5886	-2.49	3.963
	df			—	9.95	7.09	8.60	5.70	10.47	9.92
	p-value			—	0.998	0.942	0.993	0.999	0.329	0.044
**NL304**	Mean difference				—	-373	523	504.0	-693	1451 *
	t-value				—	-0.73	1.322	0.9895	-1.86	4.469
	df				—	7.38	8.97	5.95	10.74	10.00
	p-value				—	0.996	0.901	0.973	0.644	0.021
**NL305**	Mean difference					—	897	877.3	-320	1824
	t-value					—	1.613	1.3672	-0.59	3.593
	df					—	9.07	8.87	8.79	7.45
	p-value					—	0.779	0.885	0.999	0.098
**NL306**	Mean difference						—	-19.3	-121	927
	t-value						—	-0.034	-2.78	2.328
	df						—	7.53	10.60	9.05
	p-value						—	1.000	0.224	0.412
**NL307**	Mean difference							—	-119	947
	t-value							—	-2.21	1.852
	df							—	7.17	6.01
	p-value							—	0.480	0.660
**NL308**	Mean difference								—	2143 **
	t-value								—	5.746
	df								—	10.79
	p-value								—	0.003
**NL309**	Mean difference									—
	t-value									—
	df									—

Table S6: Games-Howell Post-Hoc Test – Young's Modulus

	NL301	NL302	NL303	NL304	NL305	NL306	NL307	NL308	NL309
p-value									—

Note. * $p < .05$, ** $p < .01$, *** $p < .001$
